# Health worker perspectives on barriers to delivery of routine tuberculosis diagnostic evaluation services in Uganda: a qualitative study to guide clinic-based interventions

**DOI:** 10.1186/s12913-014-0668-0

**Published:** 2015-01-22

**Authors:** Adithya Cattamanchi, Cecily R Miller, Asa Tapley, Priscilla Haguma, Emmanuel Ochom, Sara Ackerman, J Lucian Davis, Achilles Katamba, Margaret A Handley

**Affiliations:** Department of Medicine, Division of Pulmonary & Critical Care Medicine, San Francisco General Hospital, University of California San Francisco, Room 5K1, 1001 Potrero Avenue, San Francisco, California 94110 USA; Curry International Tuberculosis Center, University of California San Francisco, San Francisco, California USA; School of Medicine, University of California San Francisco, San Francisco, California USA; Department of Epidemiology and Biostatistics, University of California San Francisco, San Francisco, California USA; Department of Social and Behavioral Sciences, University of California San Francisco, San Francisco, California USA; Center for Vulnerable Populations, University of California San Francisco, San Francisco, California USA; School of MedicineMakerere University College of Health Sciences, Kampala, Uganda; Infectious Diseases Research Collaboration, Kampala, Uganda

**Keywords:** Tuberculosis, Qualitative research, Diagnosis, Barriers, Provider behavior, PRECEDE

## Abstract

**Background:**

Studies of the quality of tuberculosis (TB) diagnostic evaluation of patients in high burden countries have generally shown poor adherence to international or national guidelines. Health worker perspectives on barriers to improving TB diagnostic evaluation are critical for developing clinic-level interventions to improve guideline implementation.

**Methods:**

We conducted structured, in-depth interviews with staff at six district-level health centers in Uganda to elicit their perceptions regarding barriers to TB evaluation. Interviews were transcribed, coded with a standardized framework, and analyzed to identify emergent themes. We used thematic analysis to develop a logic model depicting health system and contextual barriers to recommended TB evaluation practices. To identify possible clinic-level interventions to improve TB evaluation, we categorized findings into predisposing, enabling, and reinforcing factors as described by the PRECEDE model, focusing on potentially modifiable behaviors at the clinic-level.

**Results:**

We interviewed 22 health center staff between February 2010 and November 2011. Participants identified key health system barriers hindering TB evaluation, including: stock-outs of drugs/supplies, inadequate space and infrastructure, lack of training, high workload, low staff motivation, and poor coordination of health center services. Contextual barrier challenges to TB evaluation were also reported, including the time and costs borne by patients to seek and complete TB evaluation, poor health literacy, and stigma against patients with TB. These contextual barriers interacted with health system barriers to contribute to sub-standard TB evaluation. Examples of intervention strategies that could address these barriers and are related to PRECEDE model components include: assigned mentors/peer coaching for new staff (targets predisposing factor of low motivation and need for support to conduct job duties); facilitated workshops to implement same day microscopy (targets enabling factor of patient barriers to completing TB evaluation), and recognition/incentives for good TB screening practices (targets low motivation and self-efficacy).

**Conclusions:**

Our findings suggest that health system and contextual barriers work together to impede TB diagnosis at health centers and, if not addressed, could hinder TB case detection efforts. Qualitative research that improves understanding of the barriers facing TB providers is critical to developing targeted interventions to improve TB care.

**Electronic supplementary material:**

The online version of this article (doi:10.1186/s12913-014-0668-0) contains supplementary material, which is available to authorized users.

## Background

Continued transmission by individuals not diagnosed and treated rapidly and appropriately is a major driver of the tuberculosis (TB) epidemic. Yet, nearly 3 million of the estimated 9 million TB cases worldwide are “missing”, either because they were not notified by health systems or not diagnosed [1]. There are two over-arching reasons for under-diagnosis: failure of patients to access TB diagnostic services and failure of providers to diagnose and treat TB among patients who do access TB diagnostic services. The latter represents a failure of the health system and, if addressed, an opportunity to increase TB case detection and treatment rates.

Studies of the quality of TB evaluation (*i.e.,* diagnostic workup of patients with symptoms suggestive of TB) in high burden countries have generally shown poor adherence to international or national guidelines. The International Standards for TB Care (ISTC), Edition 3. TB CARE I, The Hague, 2014 which have been endorsed by nearly all TB programs worldwide, recommend that at a minimum all patients in high burden countries with cough of at least 2 weeks’ duration should have at least two sputum smears examined for acid-fast bacilli (AFB) and be treated for TB if sputum AFB smear-positive. In Uganda, which is one of the 22 high TB burden countries according to the World Health Organization (WHO), we found that only 21% of patients with cough greater than 2 weeks’ duration were referred for sputum smear microscopy, 73% of patients referred completed sputum smear examination, and 71% of patients with a positive sputum examination were initiated on TB treatment [2]. Similar findings of providers not following guidelines for TB diagnosis and treatment have been reported from the public and private sector in many other high burden countries [3-9].

While previous research, including quantitative [10-12], qualitative [13-19], and mixed methods approaches [20], has assessed barriers patients face in accessing primary care centers that provide TB diagnostic services, less is known about barriers providers in these settings face in adhering to guidelines for evaluating patients for TB. The two studies evaluating provider perspectives have focused on their perceptions about patient barriers to accessing TB care [19,21]. Less well understood are the determinants of provider adherence to TB evaluation guidelines. In particular, there is increasing recognition that guideline implementation is heavily dependent on provider behavior and in order to improve the quality of care, understanding and subsequently changing provider behavior is required.

To inform the development of clinic-level interventions to improve implementation of TB evaluation guidelines, we conducted a qualitative study of front-line providers involved in TB evaluation at six primary health centers in rural Uganda. We used a theory-informed approach to elicit key determinants of the current behavior of health center staff involved in TB diagnosis and treatment. We focused on health system (clinic-level) and contextual barriers to changing provider behavior in relation to key processes associated with TB diagnosis, and used the PRECEDE framework to classify modifiable barriers as predisposing, enabling and reinforcing factors that could be targeted with clinic-level interventions [22].

## Methods

### Study setting

The study took place at six government-run Level IV health centers (HC IVs) that are located across rural Uganda and that are part of an ongoing infectious disease surveillance network. HC IVs provide preventive, general outpatient, maternity and emergency surgical care free-of-charge to a catchment population of approximately 100,000 people [23]. The HC IVs included in the network reflect the diversity of geography and malaria transmission intensity in Uganda. HC IVs have comparable staffing, which typically includes one medical officer and at least two clinical officers [24]. HC IVs are also the lowest level of the health system where quality-assured TB microscopy services are provided through the National Tuberculosis Programme.

### Recruitment and participants

All staff involved in TB diagnostic evaluation (examination of patients and referral for AFB testing, performance of sputum smear microscopy, treatment initiation, and patient navigation) at all six health centers who were present during routine quarterly site visits were asked to participate and none refused; those who had availability were interviewed, resulting in a convenience sample of available health center staff. All participants provided written informed consent, and the study was approved by institutional review boards at Makerere University (Kampala, Uganda) and the University of California, San Francisco.

### Procedures

An inter-disciplinary team including clinicians, epidemiologists, qualitative researchers and public health officials developed an interview guide focused on TB evaluation processes. The team conducted in-depth semi-structured interviews with clinic staff involved with TB evaluation, designed to elicit perspectives on TB evaluation [22,25]. We used general interview prompts to orient qualitative data descriptively in specified topic areas [26-29]. Interviews were conducted in English or in the local language during three separate visits between February 2010 and November 2011, audio-recorded and transcribed.

### Data analysis

To identify emergent themes across interviews, we developed an interview coding scheme based on “Barriers to accessing TB care by poor and vulnerable groups”, the second chapter of a 2005 WHO Report on addressing poverty in TB control [30] (Additional file 1). The data coding strategy included the following steps:

1. Several authors (MH, CM, AT) reviewed and coded transcripts independently. Individual passages were selected for inclusion into the coding template, and the primary domain and secondary sub-domains related to the quote were recorded. Some quotes had more than one code.
2. A fourth reviewer (AC) adjudicated disagreements among the primary coders.
3. Transcripts were sorted to identify thematic groupings across interviews.
4. The thematic groupings were reviewed to identify emergent themes within each domain of the coding framework and to identify quotes that best represented each domain. Emphasis was placed on describing factors affecting specific TB evaluation processes. We used the thematic analysis to develop a logic model depicting contextual and behavioral barriers to uptake of recommended TB evaluation practices (Figure 1) [25].

**Figure 1:**
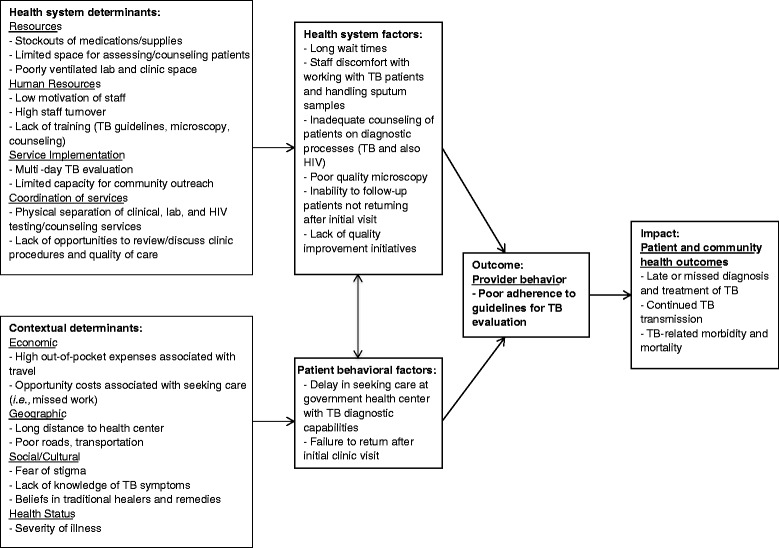
**Logic model summarizing barriers to uptake of recommended TB evaluation guidelines and resulting impact.**
5. Themes that had strong implications for understanding barriers faced at the health clinic level, and that could enhance subsequent design of health systems interventions to improve TB evaluation, were selected and categorized into predisposing, enabling and reinforcing factors as described by the PRECEDE model for health promotion (Figure 2) [22]. Predisposing factors include knowledge, motivations, and self-efficacy towards the desired behavior change; enabling factors are defined as skills and other resources that help people to adopt a recommended behavior; and reinforcing factors support and reward the desired behavior change through social support and services.

**Figure 2:**
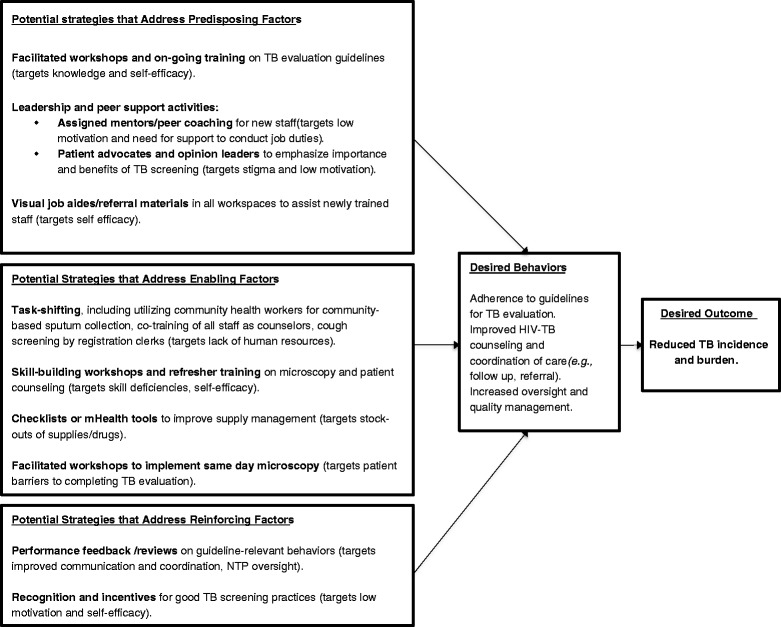
**Potential components of a clinic-level intervention to improve TB evaluation.**

## Results

Twenty-two health care providers, including 14 clinicians, five laboratory technicians, one counselor, one registration clerk, and one pharmacist, were interviewed regarding their perceptions about barriers to TB care. Three respondents (14%) were women. No one who was approached refused to participate. Results are presented according to the standardized coding framework of domains of barriers to TB diagnostic evaluation (Additional file 1). Figure 1 provides a logic model summary of how the barriers described in detail below impact the quality of TB diagnostic evaluation, leading to low TB case detection and adverse health outcomes.

### Health system barriers

#### Resources

Shortages in drugs and supplies were reported as common occurrences by clinic staff, as in these statements, “*Sometimes there’s frustrations. There are no medications. It’s just very difficult to have to work. It’s difficult, even when you want to examine the patient, sometimes you don’t have…it’s a bit difficult, but we try to improvise sometimes and make ends meet”.* When there were known medication shortages, clinic staff observed that these shortages influenced care seeking behavior, in that people stopped coming to the clinic for diagnostic services when medications were known to be in limited supply: “*It depends on whether the medications are there. If the medicines are there, this place will be completely packed by now, but because they know they’re not there, some of them will prefer to go to a private [provider]” and “…most of our patients come because of medicine, not because of a diagnosis… they come because they need something. If the medicine is not there, they’re discouraged*”. The staff also noted how drug supplies directly affect treatment: *“Sometimes we run short of drugs here.... When we run short of drugs, you put requests in time, these drugs late to come, and I mean, it interrupts treatment. You find somebody missing out on treatment for a month. It’s a very big challenge”.*

Beyond medication shortages, the lack of regular supplies (from sputum cups, slides, and gloves, to equipment like microscopes) often force staff to improvise: *“It’s difficult, even when you want to examine the patient, sometimes you don’t have [the necessary supplies]… It’s a bit difficult, but we try to improvise sometimes and make ends meet“* and *”…right now we don’t have new slides, when we get a patient we use the old slides, we recycle slides which is not good for sputum smears”,* and *“like gloves go out of stock… So you’re barehanded like this”.* Such shortages sometimes made it impossible for staff to follow recommended standards of diagnosis: *“We don’t have kits for HIV”* and “*The microscope is only one, being shared by almost all sectors. That leads to a lot of traffic jam and a delay in the service to the patients”.* Staff expressed frustration at the difficulty in receiving full stock of supplies for their clinics*: “We don’t have. We have requested. We make orders”.*

In addition to drug and supply shortages, clinic staff described that limited private space for assessing and counseling clients, lack of waiting areas, and cramped and poorly ventilated laboratory space were barriers to providing high quality care: *“Let me say again that the working space is very small -the buildings…they are not enough”*. and: *“When you’re counseling for HIV, you don’t have separate rooms…we sit here in this room. The same room, we are using for clinical, other clinical services…No privacy”*. In addition, staff reported that they were aware of how the lack of infrastructure affected their patients: *“We see over a hundred patients a day. So in bad weather like this, they have nowhere to sit. So you see them scattered there…to crowd on that veranda”* and: *“There is no privacy there. If you put a counselor there, no, it doesn’t work because everybody enters there”.*

Staff also reported that poor infrastructure contributed to the discomfort of some clinic staff in providing TB evaluation services. Clinic staff often expressed feeling unsafe levels of exposure to infection when collecting sputum from patients and when processing sputum smears in the lab: *“The TB unit, it is risky. It is just a small room…. And you see that poses a risk to the health worker”*. and*: “It poses a risk to whoever works there”*. and*: “Gloves go out of stock… So you’re barehanded like this. Mouth masks we do without”*. and*: “maybe somebody has the TB germ, this room will be full of germs”*. The staff recognized that this fear of infection sometimes negatively impacted the care that patients received: *“Some of the personnel don’t want to touch on those TB samples…I was still emphasizing on them that please the TB patients let us give them care… they need to be handled with care as we handle others”.* Last, compounding problems with supplies and infrastructure, clinicians expressed doubt as to the extent and utility of support from the National Tuberculosis Programme (NTP): *“Yes, they come (NTP staff)… I’m always seeing them around, but…we don’t know what exactly they’re doing”*. The staff also expressed concerns about the supply chain coming from the NTP: *“We don’t get communication from these fellows at the medical stores to tell us whether we don’t have drugs or we are bringing this week. So there’s a problem in information flows from medical stores”*. However, there were also some positive reports from clinic staff about NTP support: “*Recently we had complaints about understaffing the lab. They send us more tools and better technicians. They go to the laboratories and they sent to the site technicians. There are now three”.*

#### Human resources

One of the most consistent problems identified by clinic staff was the need for training, particularly smear microscopy training. High staff turnover necessitated frequent training, but staffing shortages posed difficulties to sending people for additional training. As a result, staff were often working in their jobs without completing the necessary training, as supported by these comments by lab personnel: *“My boys here – most of them were trained on the bench”* and: “*Some of us are trained, but some new staff are not trained”.*

In addition, the lack of enough staff to perform all requisite jobs led to the feeling of an untenable workload: *“We have few staff”* and: *“I’m supposed to have counselors… They are not available”* and: *“the burden is too big from a Monday to Saturday, the work load is too big”*. Sharing of duties across departments was hard to imagine given the workload felt in all areas of the clinic: *“…in the pharmacy, there is a lot of work, a lot of work… maternity is very busy, okay? Pharmacy is very busy. You can’t take anyone away”*, and: *“So during a good day the lab is very hectic”.*

Another human resource problem discussed, often tied to workload demands, was low motivation among staff: *“Then another thing is, as you know, people are demoralized”*. Some staff felt that their positions were not appropriate given their training: *“Some people who are trained…have not been promoted to their appropriate level for which they trained… It demotivates”*. Some interviewees also noted that counselors and some lab workers were volunteers and therefore could not be held accountable to a schedule: *“Sometimes they come late, and sometimes they don’t turn up.... Counseling is not included* [in staff assignment timetables]*”.*

#### Service implementation

In general, clinic staff described TB evaluation and treatment as time-consuming, putting strain on clinicians and patients alike: *“The problem is that everything associated with TB…you have to give in a lot of time… you give a sample, you wait, come tomorrow, that’s all time”*, and: *“you know, with the TB… it takes long for the results to come out”*, and: *“The challenges is mostly delays”*. Patients were often lost before completing TB evaluation, which staff attributed to the time and need for multiple visits: *“Some go in and they get the first specimen. The next day, they just don’t come back… So sometimes we miss these patients”*, and: *“The main challenge is patients not bringing the second sample”*. Clinic staff highlighted the need for more community outreach and community-based follow-up to improve service implementation, to address loss to follow-up: *“You say negative, good bye, we meet next year. Somehow somewhere there should be follow-up”, and “However much TB can be confirmed in the lab, if the follow up is not made in the community and then patients… then the lab will miss a chance of getting a TB patient”.*

#### Coordination and administration of services

Many staff identified challenges in clinic coordination that interfered with patients getting the services they needed, such that many patients disappeared from clinical care: *“the flow of patients, the problem is… they come but sometimes they don’t turn up due to lack of counseling. So they [are] maybe seen in the clinic and have a cough but don’t get enough counseling to come to the lab”*. These challenges were sometimes exacerbated by the location of clinical, lab, and HIV counseling/testing services in separate buildings: “*with the fact that the counselors are over there makes it kind of a little bit difficult to do the testing over here”.*

### Contextual barriers to TB Evaluation

#### Economic barriers

Clinic staff indicated that high costs associated with ancillary diagnostic testing, such as chest x-ray, contributed to incomplete TB diagnostic evaluation: *“you probably order for x-ray, it is a challenge… being that not all the time that we find the free x-ray films in the hospitals. So they may need to put in some money”*. The staff described how they perceived that out-of-pocket expenses accrued for patients as a result of seeking care outside of government clinics: *“It [the government site] is about five kilometers from here. So sometimes we even go to the private sector. You know, that can take some time… if they’re going to the private sector where they have to pay money, then they have to go back home and look for money”*. Staff also noted the opportunity costs for missed work, such as lost time for tending crops: *“People come from very far…Now I can do my work…I’ve started working on my garden…That will stop someone from coming to access their management of the drug*”.

Staff also reiterated that high levels of economic poverty led to low health status in their patient population, and that this made it very hard for patients to comply with treatment: “*Feeding and housing is also a big challenge to them…others go hungry… they need to eat before they take their medicine. That is very, very important*”, and *“you may find that they may not have the capacity to buy the food. So they abandon your medicine. They will not take it”.*

#### Geographic barriers

Poverty was also implicated as contributing to the burden of travelling long distances to receive care: *“You will say you will not go. You will tell me you do not have transport. ‘I’m coming from a far distance’”*, and: *“…there are some people who fail to be transported. I think this morning you saw… I guess the caretaker was the mom who was very old and who may not be having any money [for travel expenses]”*. Staff interviewees reported that many of their patients described the physical remoteness of their homes from the clinic and the tough terrain encountered during travel as principal barriers to accessing timely TB evaluation and treatment: “*People come from very far… If it’s [the] time of the rainy season … maybe there is a flood somewhere or there is a bridge which has broken”* and: *“That is a challenge, because really nobody can move the longest distance on foot.”* The staff felt that geographic remoteness combined with poverty and poor general health frequently prevent symptomatic patients from reaching the health clinics for evaluation: *“I think the problem is the long distance…And, you know, TB patients, they are weak*”. These barriers were perceived to be particularly problematic because TB evaluation requires multiple return visits: *“You can imagine I come today with my sample. You tell me that tomorrow bring the [sample]. Again you tell me to come the next day… So that kind of dosing makes some of them give up”.*

#### Social/cultural barriers

The role of stigma in the community towards patients with TB or HIV was also brought up in the interviews as a contributing factor to patients delaying TB evaluation or treatment initiation: *“The patient sometimes feels stigmatized”*, and: *“They have a stigma when you tell them, ‘You have TB’ they want to hide”* and: *“People, if they knew that in that home there is people who normally suffer from tuberculosis, the community would kind of isolate you*”. The close link between TB and HIV added to the stigma and fear of diagnosis: *“The other thing that we have still in the relationship to tuberculosis is the stigma that is related to tuberculosis and HIV”* and: *“So those two big problems will make this client almost devastated with the fear that ‘Now I have HIV. I have TB’”*. In addition, because of a fear of infection, some clinic and laboratory staff were reticent to work with patients who could have TB. Interviewed staff recognized that stigma from health providers, which often manifested as fear and ostracizing behavior, can lead to patients dropping out of the TB diagnostic process and not starting on treatment: *“…someone is told that you are going to be diagnosed for TB, they tend to scare away. That is one of the reasons…why some of them don’t come back. They fear to be diagnosed with TB and when they are clinically diagnosed…I don’t think that there is some good counseling which is done”.*

Exacerbating the challenges presented by stigma and fear, poor TB-related health literacy was additionally perceived by clinic staff as a barrier to patients seeking diagnostic testing: *“Some people take longer (to access care). They buy tablets and self-medicate, not knowing what type of cough they’re having”*, and*: “And you find that as much as we’re able to get to them on the disease, still they don’t understand—many don’t understand the spread of the disease”*. Interviewed staff also reported that poor health literacy contributed to beliefs in traditional healers and herbal remedies, which potentially delayed access to clinic-based TB evaluation: *“When they don’t complete… the reason may be that those people have not got the health education, enough health education, and now they intend to say, ‘Let’s take other types of [treatment], maybe a witch doctor’”* and: *“…the reason may be that those people have not got the health education…So other friends may divert and say, ‘No, they have bewitched you’. You know, HIV/AIDS in Africa here is related to bewitching…”.*

#### Health status barriers

There was a perception among staff at the different health centers that many patients delayed care until they were too sick to access TB diagnostic and treatment services: *“There’s no means for them to come… they were very weak, nobody to help them”*, and: *“Few people come here so those people who remain in the community they are very many…we go there in the villages find there somebody who is bedridden suspected TB but no HIV but she is there --she knows herself that she is HIV yet she is not on medicine so they are still there so many so they need sensitization”*. One interviewee observed there was a complex relationship between delaying care, worsening TB symptoms and stigma: *“The majority of TB cases that we have come across in a severe state, actually, they are almost at the dying point and one of the reasons why they are in that kind of situation is that TB has been accused”*.

### PRECEDE model for understanding barriers and developing targeted interventions

In order to identify possible clinic-level interventions to improve the quality of TB diagnostic evaluation, we categorized the health system and contextual barriers identified through staff interviews and summarized in Figure 1 into predisposing, enabling, and reinforcing factors (Figure 2). We focused on barriers that could be modified through clinic-level interventions and impact provider behavior. Key predisposing factors included low motivation of staff, stigma towards TB patients, and poor sense of self-efficacy due to time and resource constraints. Enabling factors that made it more difficult to adopt ISTC-recommended TB evaluation practices included inadequate or inconsistent microscopy and counseling skills, stock-outs of supplies and drugs and the multi-day smear examination process. Finally, reinforcing factors included insufficient or inconsistent oversight from NTP, lack of recognition of staff training or achievement, and poor communication among clinic staff.

## Discussion

We report a descriptive qualitative analysis of barriers to uptake of internationally recommended TB evaluation practices by providers at routine health centers in a high TB prevalence country. We found that health center staff perceived many of the same barriers reported previously by studies eliciting patient perspectives, including the time and costs associated with seeking and completing TB evaluation and stigma against patients with TB [10,31,32]. In addition, we identified specific health system factors that can be targeted for interventions. These ranged from behaviors relevant to the providers and other staff at the clinics (*e.g.,* lack of training, low motivation of staff, and poor coordination of services) to direct availability of resources (*e.g.,* stock-outs of supplies and drugs), both of which contribute to sub-standard TB evaluation. Next steps will focus on creating a series of targeted intervention strategies that have potential to modify provider behavior by addressing key predisposing, enabling, and reinforcing factors within this clinical setting.

Patient barriers to TB evaluation impacting initial access to TB diagnostic services have received considerable attention over the past several years. For example, previous studies have identified how long distances to health centers, direct and opportunity costs associated with seeking TB evaluation, and repeated visits to private practitioners or traditional healers delay TB evaluation [10,31]. These factors were well recognized by providers in our study and our findings indicate that providers clearly sense that these barriers continue to impact their ability to deliver high quality TB evaluation even after patients arrive at the clinic. To mitigate economic and geographic barriers, the WHO has recently endorsed a smear microscopy strategy in which two sputum specimens are collected and examined at the initial patient visit instead of over several days [33]. Our findings reinforce that TB programs in high burden countries should take steps to enable diagnosis of TB followed by initiation of treatment at a single health center visit, either through same-day microscopy or, where feasible, through use of novel semi-automated molecular diagnostic tests [34]. If implemented successfully, “test and treat” strategies are likely to have considerable impact on TB incidence and/or mortality [35].

Although same-day microscopy and semi-automated molecular tests clearly represent an important way forward to simplifying the diagnostic process, many health system barriers not unique to TB diagnosis are likely to blunt the impact of introducing new diagnostic strategies or tests if not addressed. Stock-outs of supplies and drugs and malfunctioning equipment are common at health centers in low-income countries [36]. Stock-outs not only affect the ability of staff to deliver quality care but also reinforce negative community perceptions about government health facilities, leading to patients seeking care in settings where TB testing and treatment are not routinely offered. Ministries of Health must also address barriers that may underlie the guideline concordant behaviors that will increase diagnostic service provision. These include low motivation of staff at government health facilities, partially related to concerns about the risk of acquiring TB infection from patients. Data are limited but suggest these concerns are real – health care workers in high burden countries have a 3-fold higher risk of developing TB and 6-fold higher risk of developing multidrug-resistant TB than the general population [37]. Last, poor coordination of clinic staff and services (including volunteers) was perceived as a key barrier to delivering high quality care. More attention is needed to how the timing and location of services impacts patients’ ability to receive all required care prior to completing their health center visit.

In order to build on the findings of these interviews, we propose some specific intervention strategies for application in field studies to address factors operating at multiple levels in the setting in which interventions are planned [38]. In this study we organized our qualitative findings on barriers into possible intervention components, using predisposing, enabling and reinforcing factors as intervention targets. This approach is based on a substantial literature that applies the PRECEDE model to planning health promotion activities in health care settings [39]. Our results suggest that a multi-faceted intervention to improve adherence to recommended TB evaluation practices should jointly address one of more of the following factors: 1) guideline training, leadership and peer support activities (such as mentoring/peer coaching, patient advocates, and/or opinion leaders) and visual job aides/referral materials to target knowledge, motivation of staff, stigma toward TB patients, and self-efficacy (*i.e.,* predisposing factors); 2) adequate staff training in microscopy skills, patient counseling, and care coordination; utilization of tools such as checklists or mHealth strategies to improve supply management; same-day diagnosis and treatment of TB; task-shifting, including utilizing community health workers for community-based sputum collection, co-training of all staff as counselors, implementing cough screening by registration clerks (*i.e.* enabling factors), 3) performance feedback, recognition, and incentives to increase accountability (*i.e.,* reinforcing factors). While what we presented in this paper focuses on interventions based at the health center, additional interventions that improve patient access and strengthen the larger health system are clearly needed to maximize the quality of TB evaluation services.

Our study has several potential limitations. First, the generalizability of our findings could be limited in that our study included health centers in a single country. However, the sampled health centers are from six geographically distinct districts in Uganda and provide similar services as district health centers found throughout sub-Saharan Africa. Second, our sampling of health center staff was incomplete and included only those available during site visits, limiting our ability to stratify our results by provider type. Finally, it is possible that staff were more comfortable highlighting certain barriers (such as supplies) over others (such as internal problems) that could jeopardize their standing if discussed with supervisors.

## Conclusion

In summary, we identified several substantial health system barriers to uptake of guidelines for TB evaluation at health centers in high burden countries, and confirmed the importance of contextual barriers operating at the patient and community levels. Improving the quality of TB evaluation has been shown to improve TB case detection independent of new diagnostics [2] and could amplify the impact of introducing more sensitive diagnostics as they become available. Although new diagnostics are important for increasing case detection, there is also an urgent need to identify interventions that are effective at overcoming barriers to high quality TB evaluation and that can be implemented widely at health centers in low-income countries.

## Additional file

Additional file 1:
**Classification system used to categorize identified barriers to provision of TB care, based on the WHO 2005 publication “Addressing TB and poverty in TB control” [** 30 **].**
