# Supplementary material for: Health worker perspectives on barriers to delivery of routine tuberculosis diagnostic evaluation services in Uganda: a qualitative study to guide clinic-based interventions
Source: BMC Health Serv Res. 2015 Jan 22;15:10. doi: 10.1186/s12913-014-0668-0 (PMC4307676; doi:10.1186/s12913-014-0668-0)
Supplement: Additional file 1: — Classification system used to categorize identified barriers to provision of TB care, based on the WHO 2005 publication “Addressing TB and poverty in TB control” [ 30 ]. [file 12913_2014_668_MOESM1_ESM.pdf]

Domains of barriers to TB care
I. Economic barriers
a. Health services costs<br>i. User fees for health services<br>ii. Required purchases (*i.e.* drugs, supplies, etc.)<br>b. Access to services<br>i. Transportation to health services<br>ii. Accommodation while seeking health services<br>iii. Subsistence while seeking services<br>c. Opportunity costs<br>i. Lost income while seeking health services<br>ii. Lost productivity while seeking health services<br>iii. Lost time while seeking health services
II. Geographic barriers
a. Remoteness of patients from health services<br>b. Terrain/climate impeding access to health services
III. Social/cultural barriers
a. Stigma<br>i. Disease-related (HIV, TB, etc.) stigma<br>ii. Poverty/class/gender-related stigma<br>b. Health illiteracy and related behavior<br>i. TB-related illiteracy<br>ii. General health and hygiene-related illiteracy<br>c. Lack of knowledge about health services<br>d. Traditional beliefs and practices<br>i. Traditional health-related beliefs<br>ii. Utilization of traditional health services
IV. Health status barriers
a. TB disease severity<br>b. Disease co-infection (HIV, etc.)<br>c. Diagnosing difficulty due to prevalence of other infections with overlapping symptoms (malaria, HIV, etc.)
V. Health system barriers
a. Resources<br>i. Drugs<br>ii. Supplies<br>iii. Infrastructure<br>iv. Government support<br>v. Biohazard safeguards for staff<br>b. Human resources<br>i. Training of staff<br>ii. Workload faced by staff<br>iii. Reliability and motivation of staff<br>c. Specific service implementation<br>i. TB-suspect screening<br>ii. Sample collection and evaluation<br>iii. Counseling and patient education<br>d. Coordination and administration of services<br>i. Staff allocation among services<br>ii. Clinic leadership<br>iii. Communication and coordination between services<br>iv. Patient navigation through services

v. Capacity for patient follow-up and community outreach
vi. Referrals
vii. Record keeping
